# NMR Reveals Specific Tracts within the Intrinsically Disordered Regions of the SARS-CoV-2 Nucleocapsid Protein Involved in RNA Encountering

**DOI:** 10.3390/biom12070929

**Published:** 2022-07-02

**Authors:** Letizia Pontoriero, Marco Schiavina, Sophie M. Korn, Andreas Schlundt, Roberta Pierattelli, Isabella C. Felli

**Affiliations:** 1 Magnetic Resonance Center (CERM) and Department of Chemistry “Ugo Schiff”, University of Florence, Via L. Sacconi 6, Sesto Fiorentino, 50019 Florence, Italy; pontoriero@cerm.unifi.it (L.P.); schiavina@cerm.unifi.it (M.S.); 2 Center for Biomolecular Magnetic Resonance (BMRZ), Institute for Molecular Biosciences, Johann Wolfgang Goethe-University, Max-von-Laue-Str. 9, 60438 Frankfurt, Germany; bochmann@bio.uni-frankfurt.de

**Keywords:** SARS-CoV-2, COVID-19, IDP, RNA, NMR

## Abstract

The SARS-CoV-2 nucleocapsid (N) protein is crucial for the highly organized packaging and transcription of the genomic RNA. Studying atomic details of the role of its intrinsically disordered regions (IDRs) in RNA recognition is challenging due to the absence of structure and to the repetitive nature of their primary sequence. IDRs are known to act in concert with the folded domains of N and here we use NMR spectroscopy to identify the priming events of N interacting with a regulatory SARS-CoV-2 RNA element. ^13^C-detected NMR experiments, acquired simultaneously to ^1^H detected ones, provide information on the two IDRs flanking the N-terminal RNA binding domain (NTD) within the N-terminal region of the protein (NTR, 1–248). We identify specific tracts of the IDRs that most rapidly sense and engage with RNA, and thus provide an atom-resolved picture of the interplay between the folded and disordered regions of N during RNA interaction.

## 1. Introduction

The nucleocapsid protein N of SARS-CoV-2 plays a pivotal role in the viral life cycle. The protein is organized in five different modular domains, two folded and three disordered ones, with the latter comprising almost 40% of the whole protein sequence (Supplementary Figure S1) [1,2]. It exerts various functions including packaging of genomic RNA (gRNA) inside the viral capsid [3,4,5,6,7,8] but the structural and mechanistic details of packaging remain enigmatic. The SARS-CoV-2 genome comprises a multitude of highly conserved structured *cis* regulatory RNA elements [4], which have been suggested as target sites for N in the context of packaging [9]. It is thus important to study how the disordered protein regions modulate the interaction with RNA. Recent work showed the potential of solution NMR [10,11,12,13,14,15,16,17] to describe the structural and dynamic features of different N constructs and how they interact with RNA fragments. Here we would like to explore how ^13^C detection can contribute to this field.

^13^C-NMR emerged as a key technique to study intrinsically disordered proteins (IDPs) [18]. The large chemical shift dispersion of heteronuclei (^13^C, ^15^N) is crucial to obtaining highly resolved spectra in the absence of a stable 3D structure. Solvent exchange often leads to the broadening of amide proton signals, in particular for exposed protein backbones, when approaching physiological pH and temperature. ^13^C-detected heteronuclear NMR experiments allow us to overcome this limitation. For these reasons, they constitute a valuable tool to investigate highly flexible polypeptide chains also when part of a multi-domain protein.

The contribution of the flexible regions of N to the interaction with RNA is investigated here by selecting a construct comprising the folded *N*-terminal domain NTD (44–180) and the flanking intrinsically disordered regions, IDR1 (1–43) and IDR2 (181–248). This allows us to focus on the IDRs while linked to the NTD, that is the domain deputed to bind gRNA [1]. The interaction between this N construct (1–248, referred to as N-terminal region, NTR) with RNA was studied by selecting a highly conserved *cis* element of the gRNA, namely the 5′-UTR-contained stem-loop 4 (5_SL4) [19]. This is centrally located within the 5′-UTR, has very recently been found targetable by small molecules [20] and thus represents a potential drug target to disrupt its interactions with abundant viral proteins such as N. It is described as stable [5] and is chemically versatile comprising a pentaloop, two internal loops, a bulge, and a good mix of nucleotides and types of base pairs (Supplementary Figure S1). It thus represents a bona fide example RNA for this study.

## 2. Materials and Methods

### 2.1. Protein Sample Preparation

The NTD and the NTR samples were prepared as previously described [13,21] and briefly summarized hereafter.

For the NTR construct, the gene of the N protein comprising residues 1–248 was designed based on the boundaries determined from the SARS-CoV homologue [1]. The codon-optimized gene was synthesized by Twist Bioscience and cloned into pET29b(+) vector between NdeI and XhoI restriction sites.

Uniformly ^13^C,^15^N-labeled NTR protein was expressed in *E. coli* strain BL21 (DE3) following the Marley method [22]. The cells were grown in 1 L Luria Bertani medium at 37 °C until an optical density (OD_600_) of 0.8 was reached. Then, the culture was transferred in 250 mL of labeled minimal medium supplemented with 0.25 g/L ^15^NH_4_Cl (Cambridge Isotope Laboratories) and 0.75 g/L ^13^C_6_-d-glucose (Eurisotop). After 1 h of unlabeled metabolite clearance, the culture was induced with 0.2 mM isopropyl-beta-thiogalactopyranoside (IPTG) at 16 °C for 18 h. The pellet was harvested and stored at −20 °C overnight. The cell pellet was then resuspended in 25 mM 2-amino-2-(hydroxymethyl)-1,3-propanediol (TRIS), 1.0 M NaCl, 10% glycerol, and protease inhibitor cocktail (SIGMA) at pH 8.0. Cells were disrupted by sonication and the lysate was centrifuged at 30,000× *g* for 50 min at 4 °C.

The soluble fraction was dialyzed overnight against a solution of 25 mM TRIS, pH 7.2 at 4 °C. The protein solution was then loaded on a HiTrap SP FF 5 mL column and eluted in 25 CV with a 70% gradient of 25 mM TRIS and 1.0 M NaCl. Fractions containing the protein were pooled, concentrated, and loaded on a HiLoad 16/1000 Superdex 75 pg column equilibrated with 25 mM potassium phosphate, 450 mM KCl, pH 6.5. the fractions containing the protein were pooled and concentrated using centrifugal concentrators (molecular weight cut-off 10 KDa).

The gene of the single cysteine A211C mutant of the NTR protein was synthesized by Twist Bioscience and cloned into the pET29b(+) vector between NdeI and XhoI restriction sites. Uniformly ^15^N-labeled A211C protein was expressed and purified following the same protocol used for the NTR construct, with the addition of 5 mM dithiothreitol (DTT) in the lysis and purification buffers.

The soluble fraction was dialyzed overnight against a solution of 25 mM TRIS and 5 mM DTT, pH 7.2 at 4 °C. The protein solution was then loaded on a HiTrap SP FF 5 mL column and eluted in 25 CV with a 70% gradient of 25 mM TRIS, 1.0 M NaCl, and 5 mM DTT, pH 7.2. Fractions containing the protein were pooled and concentrated to a final concentration of 25 µM.

The sequence of the NTD (44–180) was based on SARS-CoV-2 NCBI reference genome entry NC_045512.2, identical to GenBank entry MN90894 [23]. Domain boundaries for the core NTD were defined in analogy to the available NMR structure (PDB 6YI3) [10]. An *E. coli* codon-optimized DNA construct was obtained from Eurofins Genomics and sub-cloned into the pET-21-based vector pET-Trx1a, containing an *N*-terminal His_6_-tag, a thioredoxin-tag and a tobacco etch virus (TEV) cleavage site. After proteolytic TEV cleavage, the produced 14.9 kDa protein contains one artificial N-terminal residue (Gly0), before the start of the native protein sequence at Gly1 which corresponds to Gly44 in the full-length N protein sequence.

Uniformly ^15^N-labeled NTD was expressed in *E. coli* strain BL21 (DE3) in M9 minimal medium containing 1.0 g/L ^15^NH_4_Cl (Cambridge Isotope Laboratories) and 25 μg/mL kanamycin. Protein expression was induced at an OD_600_ of 0.8 with 1 mM IPTG for 18 h at room temperature. Cell pellets were resuspended in 50 mM TRIS/HCl pH 8.0, 300 mM NaCl, 10 mM imidazole, and 100 µL protease inhibitor mix (SERVA) per 1.0 L of culture. Cells were disrupted by sonication. The supernatant was cleared by centrifugation (30 min, 9000× *g*, 4 °C). The cleared supernatant was passed over a Ni^2+^-NTA gravity flow column (Sigma-Aldrich) and the His_6_-Trx-tag was cleaved overnight at 4 °C with 0.5 mg of TEV protease per 1.0 L of culture and dialyzed into fresh buffer (50 mM TRIS/HCl pH 8.0, 300 mM NaCl, 10% glycerol). TEV protease and the cleaved tag were removed via a second Ni^2+^-NTA gravity flow column, and core NTD was further purified via size exclusion on a HiLoad 16/600 SD 75 (Cytiva) in 25 mM potassium phosphate, 150 mM KCl, 2 mM Tris-(2-carboxyethyl)-phosphin (TCEP), 0.02% NaN_3_, pH 6.5. Pure NTD protein-containing fractions were determined by SDS-PAGE, pooled and concentrated using Amicon centrifugal concentrators (molecular weight cut-off of 10 kDa).

### 2.2. RNA Production

The 40 nucleotides (nt) SARS-CoV-2 genomic RNA element stem loop 4 (SL4) located within the 5′UTR (nt 86 to 125), extended 5′ by two guanine residues and 3′ by two cytidine residues, yielded the 44-nt sequence 5′-GG**GUG UGG CUG UCA CUC GGC UGC AUG CUU AGU GCA CUC ACGC** CC-3′ [19]. The DNA template for 5_SL4 was kindly provided in a HDV ribozyme vector by the COVID19-nmr consortium. The unlabeled RNA was produced by in-house optimized in vitro transcription and purified as described previously [5]. Final RNA samples were buffer-exchanged to 25 mM potassium phosphate, 150 mM KCl, pH 6.5, and sample quality, homogeneity and long-term stability were verified by native and denaturing PAGE as well as 1D-NMR experiments by means of the characteristic imino proton pattern.

### 2.3. Spin-Labeling Reaction for PRE Experiments

The A211C protein solution was purified from DTT using a PD-10 desalting column and then incubated with a ten-fold excess of S-(1-oxyl-2,2,5,5,-tetramethyl-2,5,-dihydro-1H-pyrrol-3-yl) methylmethane-sulfonothiolate (MTSL) relative to the protein concentration. The reaction was performed overnight in absence of light at 4 °C while gently stirring. Then, the unreacted spin-label was eliminated using two steps of purification with a PD-10 desalting column. The protein eluted in 25 mM TRIS and 150 mM NaCl.

To reduce MTSL and obtain the diamagnetic sample, a five-fold excess of ascorbate with respect to the protein concentration was added.

### 2.4. Protein NMR Samples

For NTR, experiments were acquired using two 500-µL-samples of 140 µM ^13^C,^15^N NTR solution in 25 mM potassium phosphate at pH 6.5, 150 mM KCl, 0.01% NaN_3_ in H_2_O with 5% D_2_O. The titration was performed in 5 mm NMR tubes. A highly concentrated batch of 5_SL4 solution in 25 mM potassium phosphate, 150 mM KCl, 0.01% NaN_3_, pH 6.5 was prepared as previously described and added to a protein solution sample in small aliquots to reach NTR:RNA ratios of 1:0.01, 1:0.025, and 1:0.05. A second identical protein sample was used to reach NTR:RNA ratios of 1:0.1, 1:0.3, and 1:0.6.

For NTD, experiments were acquired using one 500-µL-sample of 70 µM ^15^N NTD solution in 25 mM potassium phosphate at pH 6.5, 150 mM KCl, 2 mM TCEP, and 0.02% NaN_3_ in H_2_O with 5% D_2_O. A highly concentrated batch of 5_SL4 solution in 25 mM potassium phosphate, 150 mM KCl, 0.02% NaN_3_, 2 mM TCEP, and pH 6.5 was prepared as previously described and added to a protein solution sample in small aliquots to reach NTD:RNA ratios of 1:0.1, 1:0.3, 1:1.2, and 1:2.4.

### 2.5. NMR Experiments

To follow the interaction between NTR and 5_SL4, the mr_CON//HN experiment [24] was used. To complete the available assignment [13], a 3D-(H)CBCACON experiment [25] was also acquired on a 100 μM ^13^C,^15^N NTR sample.

These NMR experiments were acquired on a Bruker AVANCE NEO spectrometer operating at 700.06 MHz ^1^H, 176.05 MHz ^13^C, and 70.97 MHz ^15^N frequencies equipped with a cryogenically cooled probehead optimized for ^13^C-direct detection (TXO) at 298 K. Standard radiofrequency pulses and carrier frequencies for triple resonance experiments were used and are summarized hereafter. ^13^C pulses were given at 176.7 ppm, 55.9 ppm, and 45.7 ppm for C’, C^α^ and C^ali^ spectral regions, respectively. ^15^N pulses were given at 124.0 ppm. The ^1^H carrier was placed at 4.7 ppm. Q5- and Q3-shaped pulses [26] of durations of 300 and 231 μs, respectively, were used for ^13^C band-selective *π*/2 and *π* flip angle pulses except for the *π* pulses that should be band selective on the C*^α^* region (Q3, 1200 μs) and for the adiabatic *π* pulse to invert both C’ and C*^α^* (smoothed chirp 500 μs, 20% smoothing, 80 kHz sweep width, 11.3 kHz radio frequency field strength) [27]. Decoupling of ^1^H and ^15^N was achieved with waltz65 (100 μs) and garp4 (250 μs) decoupling sequences, respectively [26,28]. All gradients employed had a smoothed square shape.

The mr_CON//HN was acquired with an interscan delay of 1.6 s; during this delay, the HN experiment was acquired as discussed in [24]. Solvent suppression was achieved through the 3:9:19 pulse scheme [29]. For each increment of the CON experiment, acquired with 16 scans, the in-phase (IP) and antiphase (AP) components were recorded and properly combined to achieve IPAP virtual decoupling [30]. The CON spectrum was acquired with sweep widths of 5263 Hz (^13^C) × 2840 Hz (^15^N) and 1024 × 400 real points in the two dimensions, respectively. The HN spectrum was acquired with 32 scans, with sweep widths of 20869 Hz (^1^H) × 3194 Hz (^15^N) and 4096 × 400 real points in the two dimensions, respectively.

The 3D-(H)CBCACON was acquired with an interscan delay of 1 s, with 8 scans, with sweep widths of 5263 Hz (^13^C’) × 2415 Hz (^15^N) × 10,204 Hz (^13^C_ali_) and 1024 × 96 × 110 real points in the three dimensions, respectively.

To follow the interaction between NTD and 5_SL4 the 2D HN fingerprint spectra were acquired with the Fast-HSQC experimental variant [31] using a Bruker AVANCE III HD spectrometer operating at 700.17 MHz ^1^H, 176.05 MHz ^13^C, and 70.95 MHz ^15^N frequencies equipped with a quadruple-resonance cryo-probehead optimized for ^1^H-direct detection (QCI) at 298 K. The ^1^H carrier was placed at 4.7 ppm for non-selective hard pulses and the one for ^15^N at 117 ppm. The pulse scheme includes a 60 µs delay for binomial water suppression flanking the reverse INEPT step and calculated for the H^N^ central region and field strength. Decoupling of ^15^N was achieved with garp4 (250 μs) [26]. The HN experiments were acquired with an interscan delay of 1 s with 32 scans with sweep widths of 11904 Hz (^1^H) × 2412 Hz (^15^N) and 2048 × 128 real points in the two dimensions, respectively.

For the Paramagnetic Relaxation Enhancement experiments (PRE), sensitivity improvement 2D HN HSQC [32] spectra were acquired on a Bruker AVANCE NEO spectrometer operating at 900.06 (^1^H) and 91.20 (^15^N) MHz equipped with a cryogenically cooled probehead (TCI). The experiments were acquired with 32 scans, with an interscan delay of 6 s, with sweep widths of 20833 Hz (^1^H) × 3289 Hz (^15^N) and 4096 × 400 points in the two dimensions. ^15^N pulses were given at 117.0 ppm and the ^1^H carrier was placed at 4.7 ppm. Decoupling of ^15^N was achieved with garp (250 μs) decoupling sequences [26]. All gradients employed had a smoothed square shape.

### 2.6. Protein Visualization

The images and the surface potential of the proteins were created and calculated using Chimera 1.14 [33] by adding to the experimental NTD structure (PDB: 6YI3 [10]) an arbitrary conformer for IDR1 and IDR2 obtained through Flexible Meccano [34].

### 2.7. NMR Spectral Analysis

All the spectra were acquired and processed by using Bruker TopSpin 4.0.8 software. Calibration of the spectra was achieved using 4,4-dimethyl-4-silapentane-1-sulfonic acid (DSS) as a standard for ^1^H and ^13^C; ^15^N shifts were calibrated indirectly [35].

The NTR and NTD spectra were analyzed with the aid of CARA [36] and its tool NEASY [37]. All the spectra were integrated manually with NEASY taking into consideration only the well-resolved peaks. The volume of each peak, from each titration point, was divided by the volume measured in the reference spectrum acquired. The obtained ratios were plotted against the residue number. The missing values in the ratio intensity plots belong to proline residues (in the case of HN spectra), or to peaks that overlap with others, unless otherwise specified.

The Chemical Shift Perturbation (CSP) analysis was performed comparing two HN-HSQC acquired on the NTR and NTD at the same temperature and in the very same buffer (the one used for the RNA titration). The peak lists were manually inspected and only the well-resolved peaks were used to obtain the CSP values reported in the plot. The CSP values were calculated using the following equation: $CSP=\sqrt{\frac{1}{2}\left(\delta_{H}{}^{2}+0.1\cdot \delta_{N}{}^{2}\right)}$, where *δ_H_* and *δ_N_* represent the variation in the chemical shift of the ^1^H and ^15^N nuclei, respectively.

### 2.8. Electromobility Shift Assay (EMSA)

Radioactive EMSAs were performed according to [38] with the following modifications: RNA transcripts (30 pmol) were dephosphorylated using Quick CIP (NEB) following the manufacturer’s protocol and finally resuspended in H_2_O. Subsequently, 5′ end-labelling of 15 pmol SL4 RNA with [γ-^32^P]-ATP was accomplished with T4 polynucleotide kinase (NEB). Labeled RNA was separated from unincorporated [γ-^32^P]-ATP by column purification (NucAway) and adjusted with binding buffer (25 mM potassium phosphate, 150 mM KCl, pH 6.5) to 0.03 pmol/μL. A master mix containing tRNA, ^32^P-labeled SL4 RNA, and reaction buffer was prepared and then mixed with dilutions of the NTR or NTD, respectively, to achieve the indicated protein concentrations. Binding was performed for 10 min at RT in 20-μL reaction volume in the presence of 0.6 μg tRNA from baker’s yeast (Sigma), 3 nM ^32^P-labeled SL4 RNA, 25 mM potassium phosphate, 150 mM KCl, pH 6.5, and 1 mM MgCl_2_. After the addition of 3 μL loading buffer (30% glycerol, bromphenol blue, xylene cyanol), the RNP complexes were resolved by PAGE (6% polyacrylamide, 5% glycerol, and 1 × TBE) at 80 V for 75 min at RT. Gels were fixed and dried and subsequently exposed to a phosphor imager screen and visualized using a GE Typhoon laser scanner under “phosphorimager” settings.

## 3. Results and Discussion

**Figure 1:**
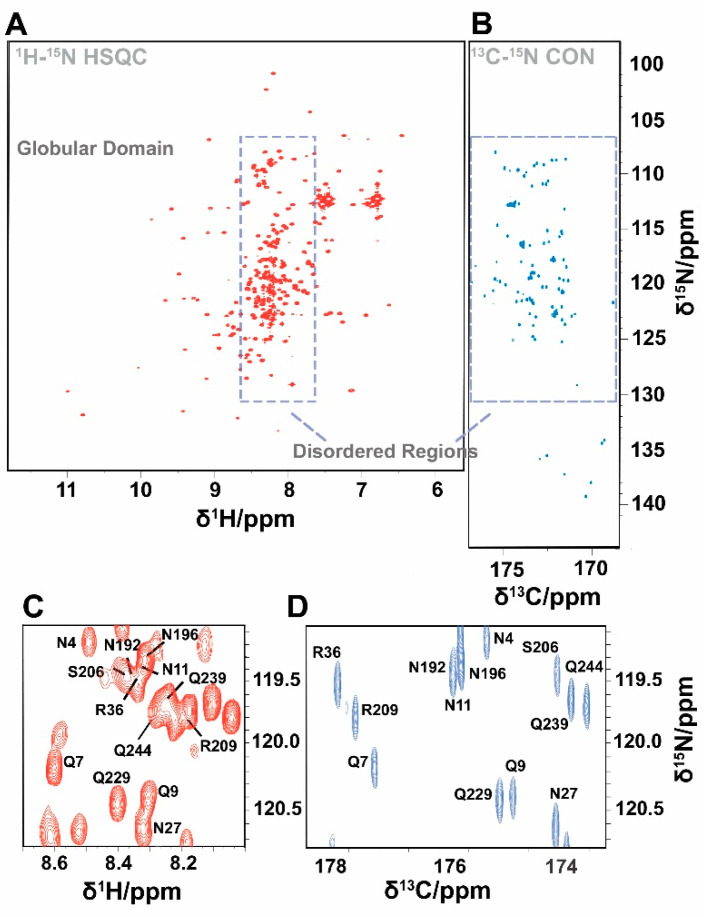
Panels A and B report the spectra obtained through the mr_CON//HN experiment. The 2D HN spectrum (**A**) shows a set of well-isolated signals deriving from the globular NTD domain as well as a number of signals, clustered in a narrow central region of the spectrum, deriving from the IDRs. The 2D CON spectrum (**B**) allows achieving the necessary resolution to investigate resonances from IDRs, including signals of proline residues. While IDR peaks fall in a very crowded region of the HN spectrum (1.1 ppm on ^1^H dimension), they are well dispersed in the CON spectrum (7.2 ppm on ^13^C dimension), as indicated by the two boxes. A zoom of a region of the two spectra centered at 120 ppm for ^15^N is reported in panels (**C**,**D**) to stress this concept.

The interaction of NTR with 5_SL4 (referred to as RNA hereafter) was studied through the ^13^C-detected ^13^C-^15^N CON (2D CON) experiment. Due to the very different structural and dynamic properties of the globular NTD domain and the flanking disordered regions, with the chosen setup, the NMR signals of the NTD are very weak or absent in the 2D CON. This allows to selectively pick up information about the disordered regions of NTR, yielding well-resolved NMR spectra, which reveal also information about seven proline residues (Figure 1). It thus provides highly complementary information to that available through a ^1^H-detected ^1^H-^15^N HSQC (2D HN) experiment. The latter allows monitoring of most of the residues belonging to the folded domain, while those of the flexible regions suffer from extensive spectral overlap or line broadening (Figure 1). The combined use of the two NMR experiments thus provides a complete picture of NTR upon interaction with RNA. The two experiments can also be collected simultaneously [24] without compromises in the quality of either of them. This experimental variant, referred to as mr_CON//HN, is particularly useful when dealing with multi-domain proteins constituted by globular domains and flexible regions. More than for time-saving, the approach is useful to achieve simultaneous snapshots of the protein which allow us to monitor the occurrence of the interaction from two different points of view. The two spectra obtained contain information about three different nuclei, one of them (^15^N) common to the two spectra. Moreover, the 2D HN can be collected with high S/N without increasing the experimental time, just exploiting the relaxation delay of the 2D CON experiment. The NMR spectra obtained through this approach on NTR are reported in Figure 1.

**Figure 2:**
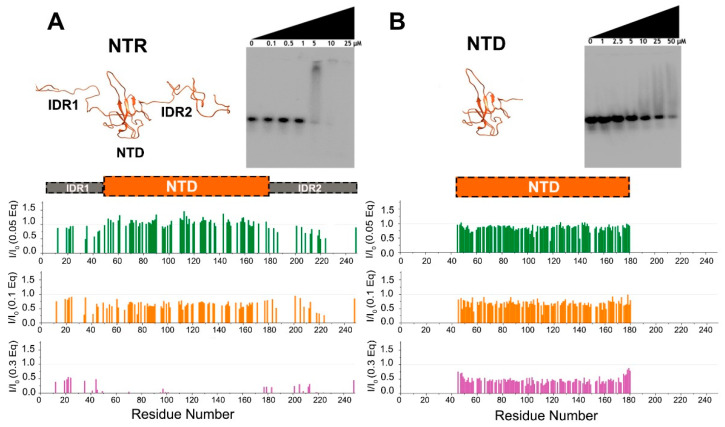
Differences in the interaction of NTR (**A**) and NTD (**B**) with 5_SL4 followed by NMR and EMSA. Upper panels show the two constructs and their different binding affinities for RNA as demonstrated by EMSA experiments. The binding of NTR to RNA occurs at a lower concentration as compared to that of NTD alone. The lower panels show plots of the HN HSQC peak intensity ratios versus residue number after the addition of increasing amounts of 5_SL4 (with equivalents as indicated) relative to protein. The structural models were obtained as described in the experimental part.

NMR spectroscopy reveals at the residue level the importance of the two disordered regions for the interaction with RNA. This is already evident when a sub-stoichiometric RNA concentration (0.05 equivalents) is added to NTR (Figure 2A). Inspection of the 2D HN spectra of NTR show variations in cross peak intensities, reported in Figure 2 as intensity ratios upon addition of increasing RNA equivalents, while shift changes are negligible (Supplementary Figure S3). In the very first points of the titration, a remarkable decrease in intensity is observed for the few resolved resonances of the HN signals from IDRs. In contrast, the signals that arise from the globular domain of the construct, seem to be less perturbed by the addition of a small RNA quantity. A further increase in RNA concentration leads to a measurable signal reduction of the NTD residues, with the complete disappearance of the signals upon the addition of 0.3 equivalents of RNA. In our experimental conditions, upon further addition of RNA, we observed liquid–liquid phase separation [11,39,40,41], not further investigated here. In contrast, the addition of RNA to the NTD (lacking the IDRs) at the same equivalent concentrations had smaller effects on line-broadening, suggesting a reduced affinity of the isolated domain (Figure 2B). This is confirmed by Electrophoretic Mobility Shift Assay (EMSA) experiments (Figure 2 and Supplementary Figure S2). The results indicate that the NTR construct has a higher affinity towards RNA compared to the NTD alone as indicated by gel shifts observed at lower concentrations. While both NTD-containing proteins show binding to RNA, the two IDRs flanking the NTD visibly increase affinity to RNA.

**Figure 3:**
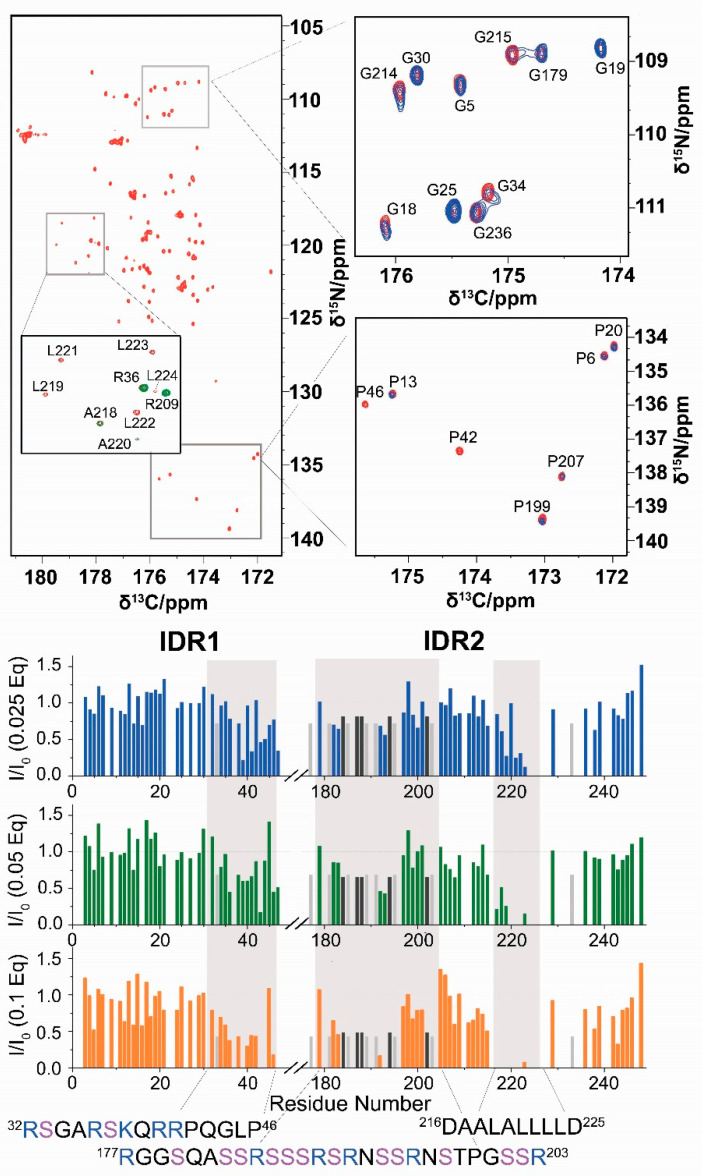
2D CON experiments reveal differential effects of RNA-binding in specific regions of IDR1 and IDR2. The CON spectrum acquired on NTR is reported in red (**top**, **left**). The inset shows the superposition of the reference spectrum with NTR upon the addition of 0.01 eq of RNA (green). The enlargements of two portions of the spectra reported on the right panels (namely, the typical Gly and Pro regions) show the spectrum acquired on the NTR upon the addition of RNA (0.1 equivalents, blue) superimposed to the spectrum acquired in the absence of RNA (reference, red). The intensity ratios of CON cross-peaks are reported in the lower panel versus the residue number; spectra were acquired simultaneously to the HN spectra. Light and dark gray bars represent the intensity ratio of the envelope of signals centered at 176.6 ppm (^13^C)–116.5 ppm (^15^N) and 174.8 ppm (^13^C)–117.9 ppm (^15^N), respectively. Gray shaded areas highlight the protein regions most perturbed upon the addition of RNA.

A zoom into the IDRs can be achieved through the analysis of the 2D CON spectrum. This allowed us to monitor most of the residues belonging to the highly flexible IDRs. As an example, Figure 3 shows the enlargement of selected portions of the 2D CON in diagnostic spectral regions such as that of glycine (top) and proline residues (bottom). Addition of 0.1 equivalents of RNA shows intensity changes for specific cross-peaks, suggesting the presence of preferred IDR sites for the interaction with RNA. Intensity ratios of the CON cross-peaks, obtained upon subsequent addition of RNA are reported versus the residue number in Figure 3. The most perturbed regions, indicated in the gray areas in Figure 3, comprise three different tracts (32–46, 177–203, and 216–225). These feature peculiar signatures in terms of amino acid composition as it often happens for interactions involving intrinsically disordered protein regions [42,43,44,45,46,47,48,49,50,51].

**Figure 4:**
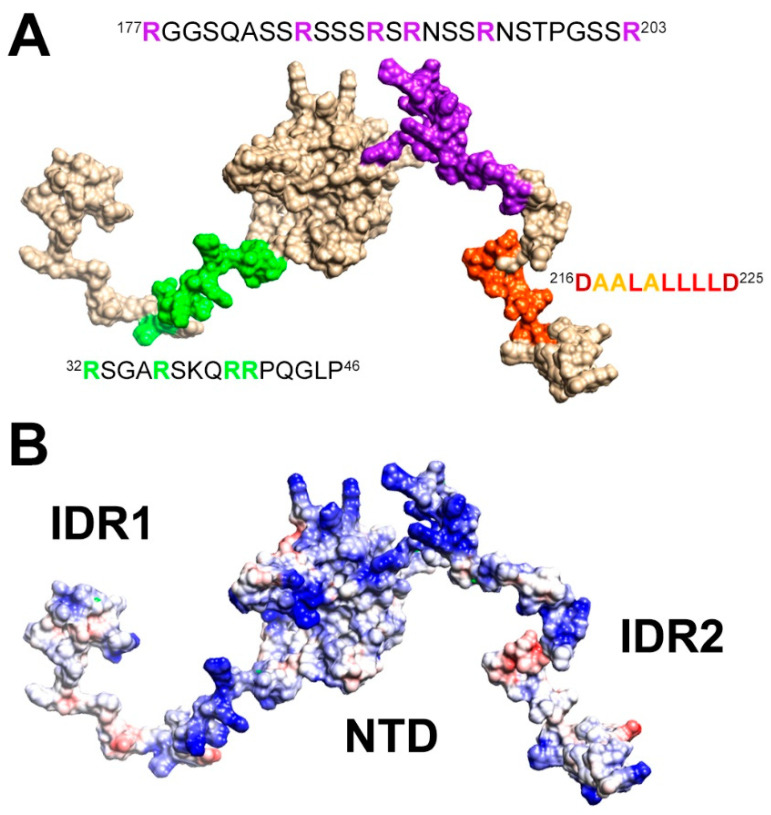
A cartoon of the NTR construct illustrating (**A**) the most perturbed regions upon the addition of RNA resulting from this study and (**B**) the large positive patch spanning both the IDRs and the globular domain. The two models were obtained as described in the experimental section.

Two of the tracts of NTR perturbed by the addition of RNA are very rich in positively charged residues: four arginine and one lysine residues in the region ^32^RSGARSKQRRPQGLP^46^, and six arginine residues in the ^177^RGGSQASSRSSSRSRNSSRNSTPGSSR^203^ region (“SR-rich region”, Supplementary Figure S1). These segments are mapped on a conformer of NTR in Figure 4A, while Figure 4B highlights the distribution of positively charged amino acids. The two tracts extend the large patch of basic residues located in the flexible, arginine-rich loop of the NTD [52], forming an extended, yet adaptable, positively charged region. These charged residues may contribute to the interaction with the RNA backbone in a priming event driven by electrostatic interactions sensed at long-distance [53]. Notably, these two regions are likely targets of regulatory post-translational modifications, such as the phosphorylation of the serine residues within the SR-rich portion that alters the overall charge of this tract (Supplementary Figure S4) [11,54].

The third region that is perturbed by the addition of RNA (216–225) has completely different properties. This region possesses a peculiar amino acid composition (^216^DAALALLLLD^225^, Figure 4A) and the NMR signals of the hydrophobic residues are weak, likely due to a helical propensity of this segment, which is reflected in signal broadening due to exchange with the protein-free conformation. Indeed, sequence-specific assignment of resonances in this region posed challenges to different NMR approaches before [13,14,16]. We obtained the assignment of the resonances belonging to these residues by exploiting a 3D (H)CBCACON experiment (Figure S5), thus extending the previously obtained sequence-specific assignment [13].

Differently from the two arginine-rich regions involved in the interaction with RNA (32–46 and 177–203), the 216–225 region does not present positively charged amino acids but has a highly hydrophobic nature resulting from branched-chain amino acids such as leucine [thus referred to as the poly-leucine (poly-L) region]. This hydrophobic stretch of 8 amino acids flanked by two negatively charged residues (Asp 216 and Asp 225) is likely to be engaged in transient interactions with other portions of NTR. A comparison of chemical shifts observed for the isolated NTD with those of the same nuclei in the NTR construct supports this hypothesis, and the insertion of a spin-label at position 211 indeed confirms a cross-talk between the IDR and the NTD domain (Supplementary Figure S6). Of note, the potency of the poly-L stretch to mediate protein-protein interactions has very recently been manifested in its complex with the SARS-CoV-2 nsp3 Ubl domain, while, interestingly, this interaction competes with RNA-binding of N [17]. Our data support this picture in which the poly-L region serves as an interactive hub. From our data, the observed intensity changes upon the addition of RNA in the poly-L region could derive both from direct interactions with RNA as well as from weak/fuzzy intra-molecular interactions involving different domains of NTR that are disrupted by the interaction with RNA. The latter effect might alter the dynamic properties of NTR and account for the slight increase in relative signal intensities of the globular domain observed when sub-stoichiometric amounts of RNA are added (Figure 2A). Judging by our and the previous data [17], the poly-L region might act as a regulatory motif that, within N, releases the NTD in presence of RNA and/or guides the protein to functionally relevant RNP complexes via protein-protein interactions.

Summarizing, the present results indicate that electrostatics is the main driving force for molecular recognition and the arginine-rich regions, that were found to be perturbed at the early stages of the titration, are key players to promote binding with the negatively charged RNA backbone [55,56]. Interactions between disordered protein regions with complementary charges have indeed been shown to lead to high-affinity complexes [50]. The involvement of the flexible linkers is however not limited to the arginine-rich regions but also includes the poly-L region preset in IDR2. Altogether, this suggests a complex interplay between various parts of the NTR construct.

The experimental investigation of the highly dynamic properties of N is by no means a trivial task but is of crucial importance to identifying novel approaches to interfere with SARS-CoV-2. Several insights have been recently obtained on its dynamic heterogeneity [16], on the key role of the SR-rich [11] region, on the interaction with a viral chaperone, nsp3 [17]. The interaction of NTD with different RNA fragments has been studied [10,15]. In many cases, detection of NMR signals required the use of short constructs [11,14,16,17] or changes in pH and T [16]. Increasing the complexity of the system [12] revealed very interesting insights although at the expense of residue-resolved information on the disordered regions. The proposed approach offers a tool to overcome these limitations and observe in a clean way highly flexible disordered regions within multi-domain protein constructs. As an example, the 210–248 region that comprises 56% of the IDR2 residues is challenging to observe unless smaller fragments are studied, but deletion of this region from the full-length protein has been shown to significantly alter protein function [41]. It is worth noting that this portion (219–230) shares many physicochemical properties with nucleocapsids from related coronaviruses [1,2,3].

## 4. Conclusions

In conclusion, ^13^C-detected NMR experiments such as the 2D CON allow us to access residue-resolved information on IDRs also when part of a multi-domain protein. They can be added to any high-resolution investigation performed through NMR, often based on the analysis of 2D HN NMR spectra only. The mr_CON//HN approach allows their simultaneous acquisition, providing a complete picture at residue level not only for the flexible regions but at the same time for the globular NTD domain. This complementary information is highly valuable as it reflects all components in their native context.

The NMR data, supported by EMSA data, demonstrated that the flanking disordered regions of the SARS-CoV-2 NTD initiate and enhance the binding of the protein to RNA. They revealed specific tracts of the IDRs involved in the interaction within a multi-domain, cleavage prone, structurally and dynamically complex protein as NTR is.

This represents a first step necessary to unravel the detailed molecular determinants of the N protein for specific RNA encountering and subsequent complex formation, e.g., during viral genome packaging. It paves the way for further studies with increasingly complex protein constructs, ultimately with the full-length protein, as well as with other relevant elements of the SARS-CoV-2 RNA.

## Data Availability

The data presented in this study are available upon request from the corresponding authors.

## Supplementary Materials

The following supporting information can be downloaded at: https://www.mdpi.com/article/10.3390/biom12070929/s1, Figure S1: molecular components used in this study [57,58]; Figure S2: Triplicates of EMSA gels. Figure S3: 2D HN spectra of NTR upon addiction of RNA; Figure S4: NetPhos results [59]; Figure S5: 3D-(H)CBCACON strips; Figure S6: CSP and PRE results. It also includes the additional references.

## References

[1] Chang C.K., Hou M.H., Chang C.F., Hsiao C.D., Huang T.H. (2014). The SARS Coronavirus Nucleocapsid Protein—Forms and Functions. Antivir. Res..

[2] Giri R., Bhardwaj T., Shegane M., Gehi B.R., Kumar P., Gadhave K., Oldfield C.J., Uversky V.N. (2021). Understanding COVID-19 via Comparative Analysis of Dark Proteomes of SARS-CoV-2, Human SARS and Bat SARS-like Coronaviruses. Cell. Mol. Life Sci..

[3] Chang C.-K., Hsu Y.-L., Chang Y.-H., Chao F.-A., Wu M.-C., Huang Y.-S., Hu C.-K., Huang T.-H. (2009). Multiple Nucleic Acid Binding Sites and Intrinsic Disorder of Severe Acute Respiratory Syndrome Coronavirus Nucleocapsid Protein: Implications for Ribonucleocapsid Protein Packaging. J. Virol..

[4] Rangan R., Zheludev I.N., Hagey R.J., Pham E.A., Wayment-Steele H.K., Glenn J.S., Das R. (2020). RNA Genome Conservation and Secondary Structure in SARS-CoV-2 and SARS-Related Viruses: A First Look. RNA.

[5] Wacker A., Weigand J.E., Akabayov S.R., Altincekic N., Bains J.K., Banijamali E., Binas O., Castillo-Martinez J., Cetiner E., Ceylan B. (2020). Secondary Structure Determination of Conserved SARS-CoV-2 RNA Elements by NMR Spectroscopy. Nucleic Acids Res..

[6] Cao C., Cai Z., Xiao X., Rao J., Chen J., Hu N., Yang M., Xing X., Wang Y., Li M. (2021). The Architecture of the SARS-CoV-2 RNA Genome inside Virion. Nat. Commun..

[7] de Tavares R.C.A., Mahadeshwar G., Wan H., Huston N.C., Pyle A.M. (2021). The Global and Local Distribution of RNA Structure throughout the SARS-CoV-2 Genome. J. Virol..

[8] Bai Z., Cao Y., Liu W., Li J. (2021). The SARS-CoV-2 Nucleocapsid Protein and Its Role in Viral Structure, Biological Functions, and a Potential Target for Drug or Vaccine Mitigation. Viruses.

[9] Iserman C., Roden C.A., Boerneke M.A., Sealfon R.S.G., McLaughlin G.A., Jungreis I., Fritch E.J., Hou Y.J., Ekena J., Weidmann C.A. (2020). Genomic RNA Elements Drive Phase Separation of the SARS-CoV-2 Nucleocapsid. Mol. Cell.

[10] Dinesh D.C., Chalupska D., Silhan J., Koutna E., Nencka R., Veverka V., Boura E. (2020). Structural Basis of RNA Recognition by the SARS-CoV-2 Nucleocapsid Phosphoprotein. PLoS Pathog..

[11] Savastano A., de Opakua A.I., Rankovic M., Zweckstetter M. (2020). Nucleocapsid Protein of SARS-CoV-2 Phase Separates into RNA-Rich Polymerase-Containing Condensates. Nat. Commun..

[12] Forsythe H.M., Rodriguez Galvan J., Yu Z., Pinckney S., Reardon P., Cooley R.B., Zhu P., Rolland A.D., Prell J.S., Barbar E. (2021). Multivalent Binding of the Partially Disordered SARS-CoV-2 Nucleocapsid Phosphoprotein Dimer to RNA. Biophys. J..

[13] Schiavina M., Pontoriero L., Uversky V.N., Felli I.C., Pierattelli R. (2021). The Highly Flexible Disordered Regions of the SARS-CoV-2 Nucleocapsid N Protein within the 1–248 Residue Construct: Sequence-Specific Resonance Assignments through NMR. Biomol. NMR Assign..

[14] Guseva S., Perez L.M., Camacho-Zarco A., Bessa L.M., Salvi N., Malki A., Maurin D., Blackledge M. (2021). ^1^H, ^13^C and ^15^N Backbone Chemical Shift Assignments of the N-Terminal and Central Intrinsically Disordered Domains of SARS-CoV-2 Nucleoprotein. Biomol. NMR Assign..

[15] Caruso Í.P., Sanches K., Da Poian A.T., Pinheiro A.S., Almeida F.C.L. (2021). Dynamics of the SARS-CoV-2 Nucleoprotein N-Terminal Domain Triggers RNA Duplex Destabilization. Biophys. J..

[16] Redzic J.S., Lee E., Born A., Issaian A., Henen M.A., Nichols P.J., Blue A., Hansen K.C., D’Alessandro A., Vögeli B. (2021). The Inherent Dynamics and Interaction Sites of the SARS-CoV-2 Nucleocapsid N-Terminal Region. J. Mol. Biol..

[17] Bessa L.M., Guseva S., Camacho-Zarco A.R., Salvi N., Maurin D., Perez L.M., Botova M., Malki A., Nanao M., Jensen M.R. (2022). The Intrinsically Disordered SARS-CoV-2 Nucleoprotein in Dynamic Complex with Its Viral Partner Nsp3a. Sci. Adv..

[18] Felli I.C., Pierattelli R. (2022). ^13^C Direct Detected NMR for Challenging Systems. Chem. Rev..

[19] Vögele J., Ferner J.-P., Altincekic N., Bains J.K., Ceylan B., Fürtig B., Grün J.T., Hengesbach M., Hohmann K.F., Hymon D. (2021). ^1^H, ^13^C, ^15^N and ^31^P Chemical Shift Assignment for Stem-Loop 4 from the 5′-UTR of SARS-CoV-2. Biomol. NMR Assign..

[20] Sreeramulu S., Richter C., Berg H., Wirtz Martin M.A., Ceylan B., Matzel T., Adam J., Altincekic N., Azzaoui K., Bains J.K. (2021). Exploring the Druggability of Conserved RNA Regulatory Elements in the SARS-CoV-2 Genome. Angew. Chem. Int. Ed..

[21] Altincekic N., Korn S.M., Qureshi N.S., Dujardin M., Ninot-Pedrosa M., Abele R., Abi Saad M.J., Alfano C., Almeida F.C.L., Alshamleh I. (2021). Large-Scale Recombinant Production of the SARS-CoV-2 Proteome for High-Throughput and Structural Biology Applications. Front. Mol. Biosci..

[22] Marley J., Lu M., Bracken C. (2001). A Method for Efficient Isotopic Labeling of Recombinant Proteins. J. Biomol. NMR.

[23] Wu F., Zhao S., Yu B., Chen Y.-M., Wang W., Song Z.-G., Hu Y., Tao Z.-W., Tian J.-H., Pei Y.-Y. (2020). A New Coronavirus Associated with Human Respiratory Disease in China. Nature.

[24] Schiavina M., Murrali M.G., Pontoriero L., Sainati V., Kümmerle R., Bermel W., Pierattelli R., Felli I.C. (2019). Taking Simultaneous Snapshots of Intrinsically Disordered Proteins in Action. Biophys. J..

[25] Bermel W., Bertini I., Csizmok V., Felli I.C., Pierattelli R., Tompa P. (2009). H-Start for Exclusively Heteronuclear NMR Spectroscopy: The Case of Intrinsically Disordered Proteins. J. Magn. Reson..

[26] Emsley L., Bodenhausen G. (1992). Optimization of Shaped Selective Pulses for NMR Using a Quaternion Description of Their Overall Propagators. J. Magn. Reson..

[27] Böhlen J.M., Bodenhausen G. (1993). Experimental Aspects of Chirp NMR Spectroscopy. J. Magn. Reson. Ser. A.

[28] Geen H., Freeman R. (1991). Band-Selective Radiofrequency Pulses. J. Magn. Reson..

[29] Piotto M., Saudek V., Sklenar V. (1992). Gradient-Tailored Excitation for Single-Quantum NMR Spectroscopy of Aqueous Solutions. J. Biomol. NMR.

[30] Felli I.C., Pierattelli R. (2015). Spin-State-Selective Methods in Solution- and Solid-State Biomolecular ^13^C NMR. Prog. Nucl. Magn. Reson. Spectrosc..

[31] Mori S., Abeygunawardana C., Johnson M.O., Vanzijl P.C.M. (1995). Improved Sensitivity of HSQC Spectra of Exchanging Protons at Short Interscan Delays Using a New Fast HSQC (FHSQC) Detection Scheme That Avoids Water Saturation. J. Magn. Reson. Ser. B.

[32] Palmer A.G., Cavanagh J., Wright P.E., Rance M. (1991). Sensitivity Improvement in Proton-Detected Two-Dimensional Heteronuclear Correlation NMR Spectroscopy. J. Magn. Reson..

[33] Pettersen E.F., Goddard T.D., Huang C.C., Couch G.S., Greenblatt D.M., Meng E.C., Ferrin T.E. (2004). UCSF Chimera: A Visualization System for Exploratory Research and Analysis. J. Comput. Chem..

[34] Ozenne V., Bauer F., Salmon L., Huang J.R., Jensen M.R., Segard S., Bernadó P., Charavay C., Blackledge M. (2012). Flexible-Meccano: A Tool for the Generation of Explicit Ensemble Descriptions of Intrinsically Disordered Proteins and Their Associated Experimental Observables. Bioinformatics.

[35] Markley J.L., Bax A., Arata Y., Hilbers C.W., Kaptein R., Sykes B.D., Wright P.E., Wuethrich K. (1998). Recommendations for the Presentation of NMR Structures of Proteins and Nucleic Acids. Pure Appl. Chem..

[36] Keller R. (2004). The Computer Aided Resonance Assignment Tutorial.

[37] Bartels C., Xia T.H., Billeter M., Güntert P., Wüthrich K. (1995). The Program XEASY for Computer-Supported NMR Spectral Analysis of Biological Macromolecules. J. Biomol. NMR.

[38] Ryder S.P., Recht M.I., Williamson J.R. (2008). Quantitative Analysis of Protein-RNA Interactions by Gel Mobility Shift. Methods Mol. Biol..

[39] Perdikari T.M., Murthy A.C., Ryan V.H., Watters S., Naik M.T., Fawzi N.L. (2020). SARS-CoV-2 Nucleocapsid Protein Phase-separates with RNA and with Human HnRNPs. EMBO J..

[40] Cubuk J., Alston J.J., Incicco J.J., Singh S., Stuchell-Brereton M.D., Ward M.D., Zimmerman M.I., Vithani N., Griffith D., Wagoner J.A. (2021). The SARS-CoV-2 Nucleocapsid Protein Is Dynamic, Disordered, and Phase Separates with RNA. Nat. Commun..

[41] Lu S., Ye Q., Singh D., Cao Y., Diedrich J.K., Yates J.R., Villa E., Cleveland D.W., Corbett K.D. (2021). The SARS-CoV-2 Nucleocapsid Phosphoprotein Forms Mutually Exclusive Condensates with RNA and the Membrane-Associated M Protein. Nat. Commun..

[42] Tompa P., Fuxreiter M. (2008). Fuzzy Complexes: Polymorphism and Structural Disorder in Protein-Protein Interactions. Trends Biochem. Sci..

[43] Mittag T., Kay L.E., Forman-Kay J.D. (2009). Protein Dynamics and Conformational Disorder in Molecular Recognition. J. Mol. Recognit..

[44] Kurzbach D., Schwarz T.C., Platzer G., Höfler S., Hinderberger D., Konrat R. (2014). Compensatory Adaptations of Structural Dynamics in an Intrinsically Disordered Protein Complex. Angew. Chem. Int. Ed..

[45] Habchi J., Tompa P., Longhi S., Uversky V.N. (2014). Introducing Protein Intrinsic Disorder. Chem. Rev..

[46] Fuxreiter M., Tóth-Petróczy Á., Kraut D.A., Matouschek A., Matouschek A.T., Lim R.Y.H., Xue B., Kurgan L., Uversky V.N. (2014). Disordered Proteinaceous Machines. Chem. Rev..

[47] Contreras-Martos S., Piai A., Kosol S., Varadi M., Bekesi A., Lebrun P., Volkov A.N., Gevaert K., Pierattelli R., Felli I.C. (2017). Linking Functions: An Additional Role for an Intrinsically Disordered Linker Domain in the Transcriptional Coactivator CBP. Sci. Rep..

[48] Arbesú M., Iruela G., Fuentes H., Teixeira J.M.C., Pons M. (2018). Intramolecular Fuzzy Interactions Involving Intrinsically Disordered Domains. Front. Mol. Biosci..

[49] Spreitzer E., Usluer S., Madl T. (2020). Probing Surfaces in Dynamic Protein Interactions. J. Mol. Biol..

[50] Sottini A., Borgia A., Borgia M.B., Bugge K., Nettels D., Chowdhury A., Heidarsson P.O., Zosel F., Best R.B., Kragelund B.B. (2020). Polyelectrolyte Interactions Enable Rapid Association and Dissociation in High-Affinity Disordered Protein Complexes. Nat. Commun..

[51] Murrali M.G., Felli I.C., Pierattelli R. (2020). Adenoviral E1A Exploits Flexibility and Disorder to Target Cellular Proteins. Biomolecules.

[52] Clarkson M.W., Lei M., Eisenmesser E.Z., Labeikovsky W., Redfield A., Kern D. (2009). Mesodynamics in the SARS Nucleocapsid Measured by NMR Field Cycling. J. Biomol. NMR.

[53] Das R.K., Pappu R.V. (2013). Conformations of Intrinsically Disordered Proteins Are Influenced by Linear Sequence Distributions of Oppositely Charged Residues. Proc. Natl. Acad. Sci. USA.

[54] Carlson C.R., Asfaha J.B., Ghent C.M., Howard C.J., Hartooni N., Safari M., Frankel A.D., Morgan D.O. (2020). Phosphoregulation of Phase Separation by the SARS-CoV-2 N Protein Suggests a Biophysical Basis for Its Dual Functions. Mol. Cell.

[55] Calabretta S., Richard S. (2015). Emerging Roles of Disordered Sequences in RNA-Binding Proteins. Trends Biochem. Sci..

[56] Järvelin A.I., Noerenberg M., Davis I., Castello A. (2016). The New (Dis)Order in RNA Regulation. Cell Commun. Signal..

[57] Popenda M., Szachniuk M., Antczak M., Purzycka K.J., Lukasiak P., Bartol N., Blazewicz J., Adamiak R.W. (2012). Automated 3D Structure Composition for Large RNAs. Nucleic Acids Res..

[58] Hofacker I.L. (2003). Vienna RNA Secondary Structure Server. Nucleic Acids Res..

[59] Blom N., Sicheritz-Pontén T., Gupta R., Gammeltoft S., Brunak S. (2004). Prediction of Post-Translational Glycosylation and Phosphorylation of Proteins from the Amino Acid Sequence. Proteomics.

