# Supplementary material for: NMR Reveals Specific Tracts within the Intrinsically Disordered Regions of the SARS-CoV-2 Nucleocapsid Protein Involved in RNA Encountering
Source: Biomolecules. 2022 Jul 2;12(7):929. doi: 10.3390/biom12070929 (PMC9312987; doi:10.3390/biom12070929)
Supplement: Supplementary file 1 [file biomolecules-12-00929-s001.zip › biomolecules-1782046-supplementary.pdf]

NMR Reveals Specific Tracts within the Intrinsically Disordered Regions of the SARS-CoV-2 Nucleocapsid Protein Involved in RNA Encountering

Letizia Pontoriero [1,†], Marco Schiavina [1,†], Sophie M. Korn [2,†], Andreas Schlundt [2,*], Roberta Pierattelli [1,*] and Isabella C. Felli [1,*]

1 Magnetic Resonance Center (CERM) and Department of Chemistry "Ugo Schiff", University of Florence, Via L. Sacconi 6, 50019 Sesto Fiorentino (Florence), Italy; pontoriero@cerm.unifi.it (L.P.); schiavina@cerm.unifi.it (M.S.)

2 Institute for Molecular Biosciences, Center for Biomolecular Magnetic Resonance (BMRZ), Johann Wolfgang Goethe-University, Max-von-Laue-Str. 9, 60438 Frankfurt a. M., Germany; bochmann@bio.uni-frankfurt.de (S.M.K.)

* Correspondence: schlundt@bio.uni-frankfurt.de (A.S.); roberta.pierattelli@unifi.it (R.P.); felli@cerm.unifi.it (I.C.F.)

† These authors contributed equally to the work.

Supplementary Figures

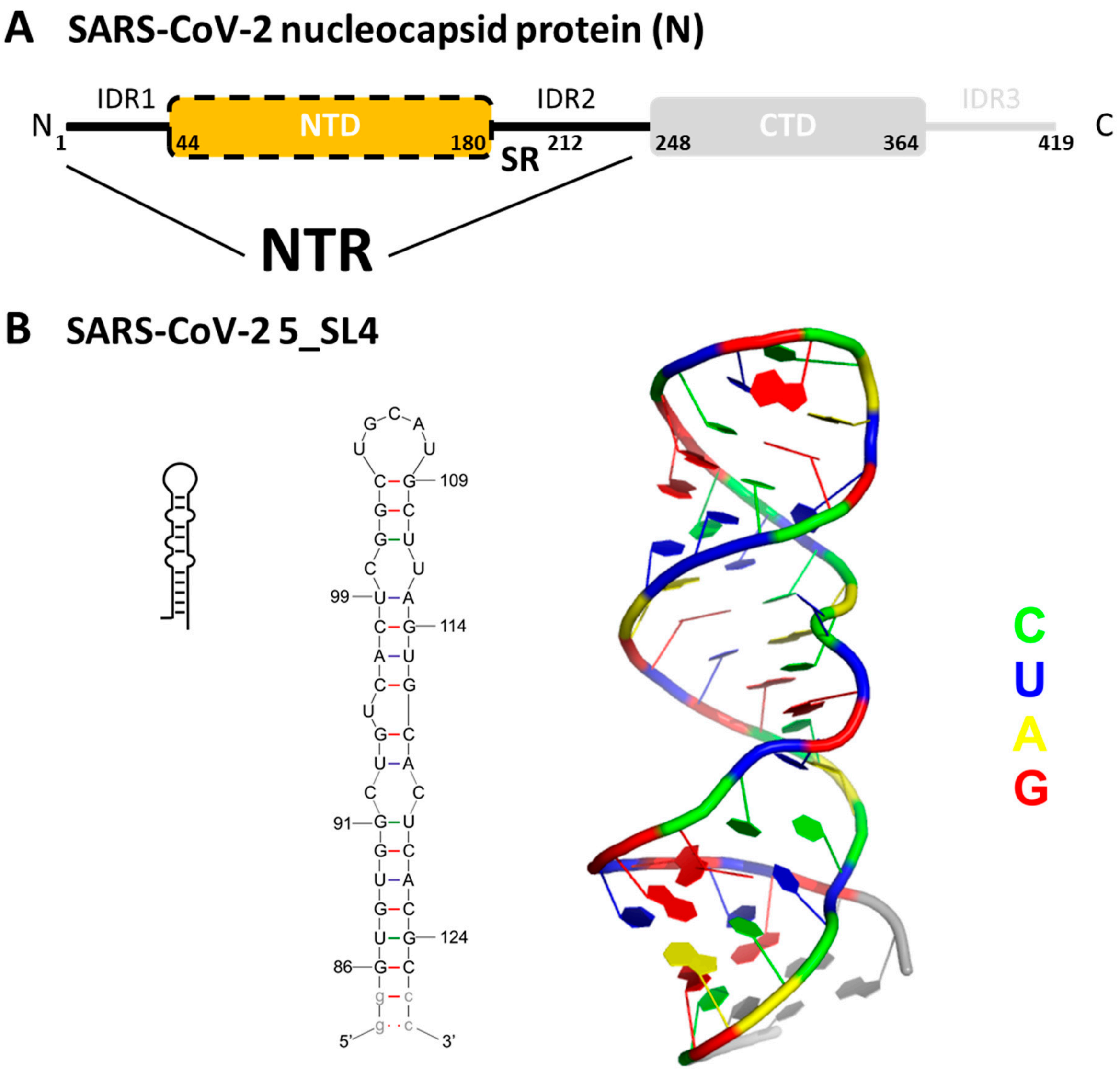

Figure S1: Overview of the molecular components used in this study. A) Domains organization of the SARS-CoV-2 full-length N protein. Numbers indicate the herein considered boundaries between IDRs and globular domains. Grey-shaded regions beyond residue 248 are not part of this study. The IDR2-embedded SR region is shown for convenience. B) Secondary structure and model of 5_SL4 as obtained from RNAcomposer [57] and based on RNAfold [58]. Color-coded nucleotides are used to visualize the base distribution. The secondary structure is based on [3] including genomic numbering. Grey bases indicate artificial nucleotides in the construct used for RNA in vitro transcription.

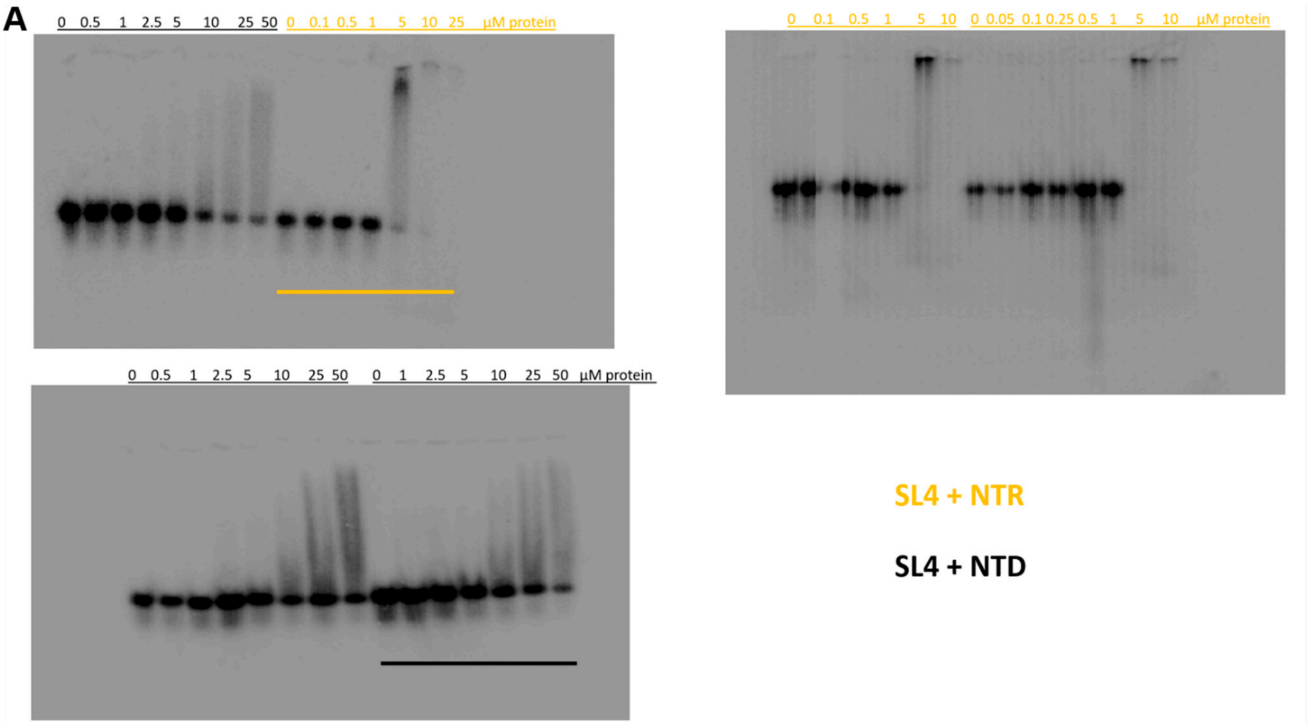

Figure S2: Interaction between NTR and NTD with SL4 RNA. Triplicates of EMSAs as shown in main text Figure 2. Presented are uncropped images as obtained from the Phosphoimager (see Methods). Concentrations of proteins added to SL4 RNA are given above respective lanes (orange, NTR and black, NTD). Bars indicate the replicates that have been used for the main figure panel, respectively.

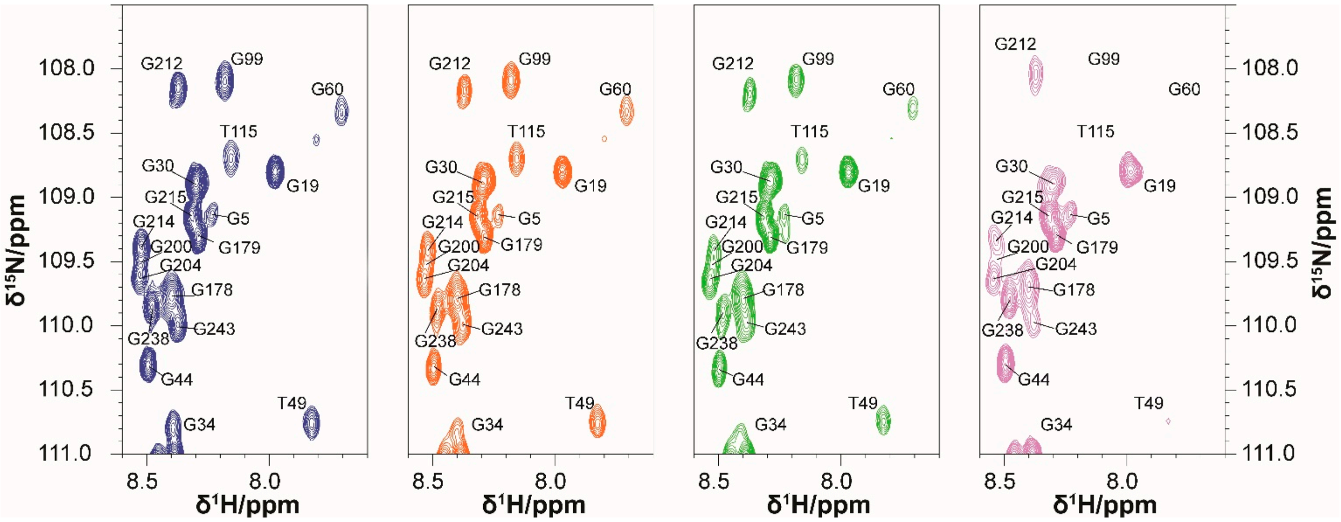

Figure S3: Series of 2D HN spectra of NTR upon addition of different RNA equivalents are reported in Blue (Reference), Orange (0.05 RNA equivalents), Green (0.10 RNA equivalents) and Pink (0.30 RNA equivalents). The overall picture displays negligible chemical shift perturbations, but a drastic decrease in intensity (see e.g., G99 and T49).

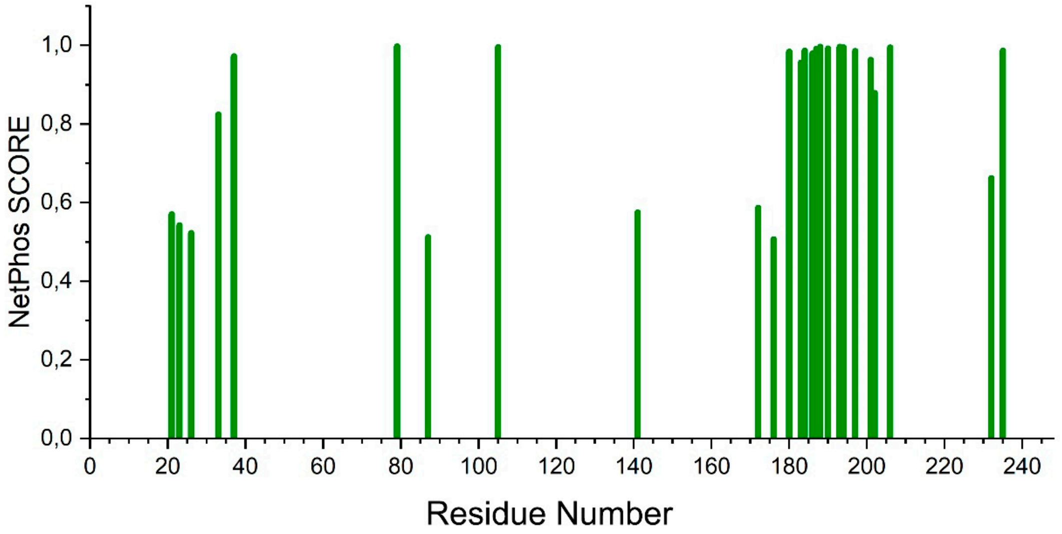

Figure S4: NetPhos [59] results for the serine residues of the NTR construct. Only the serine residues displaying a NetPhos score higher than 0.5 are reported.

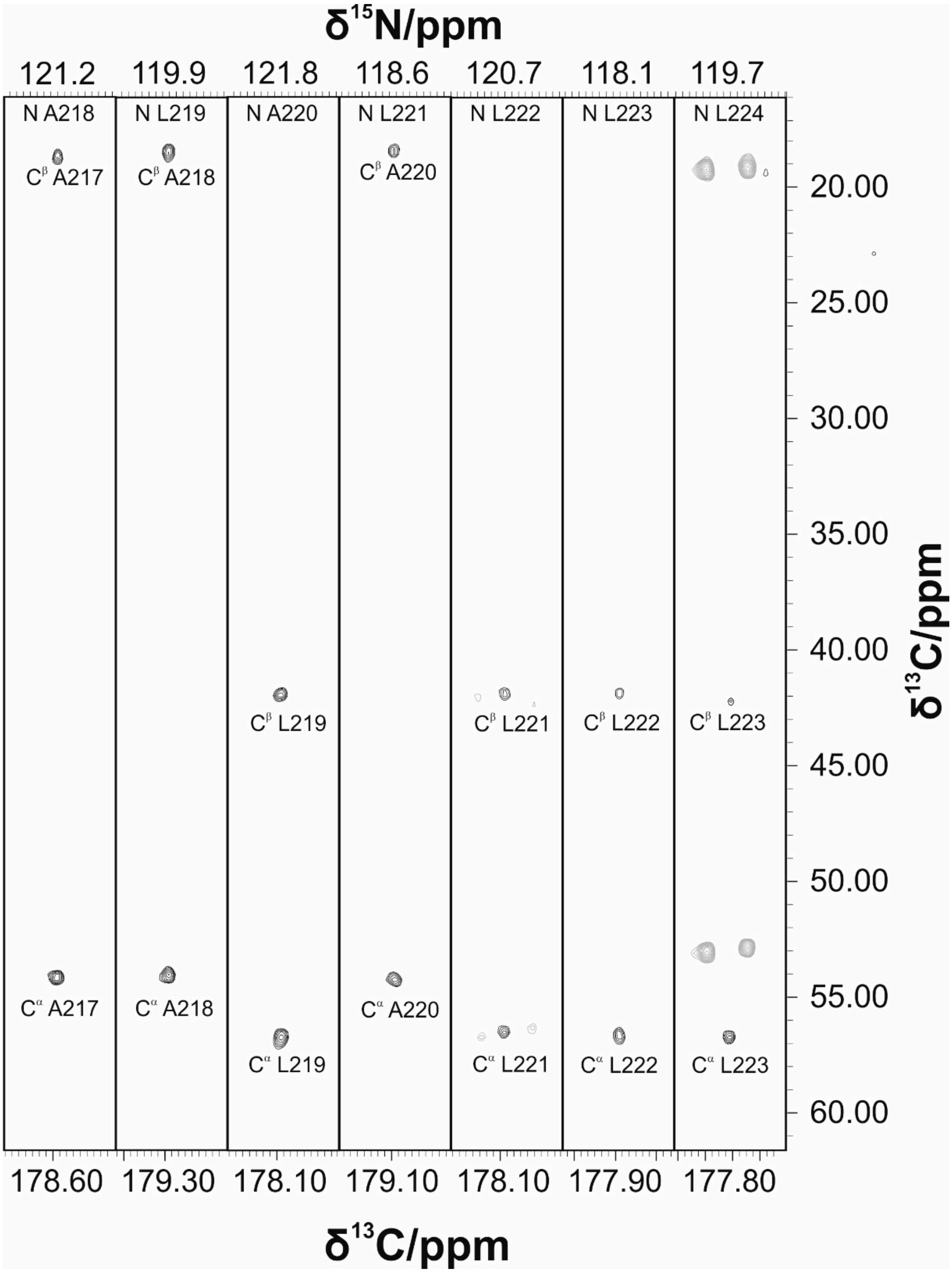

Figure S5: Strips extracted from the 3D (H)CBCACON used to identify the resonances of the poly-Leucine stretch. ^{13}C-Resonances of the fourth leucine residue in the four-amino acid sequence (^{221}LLLL^{224}) were identified in the 2D CACO and 2D CBCACO experiments and assigned to Leu 224 by exclusion.

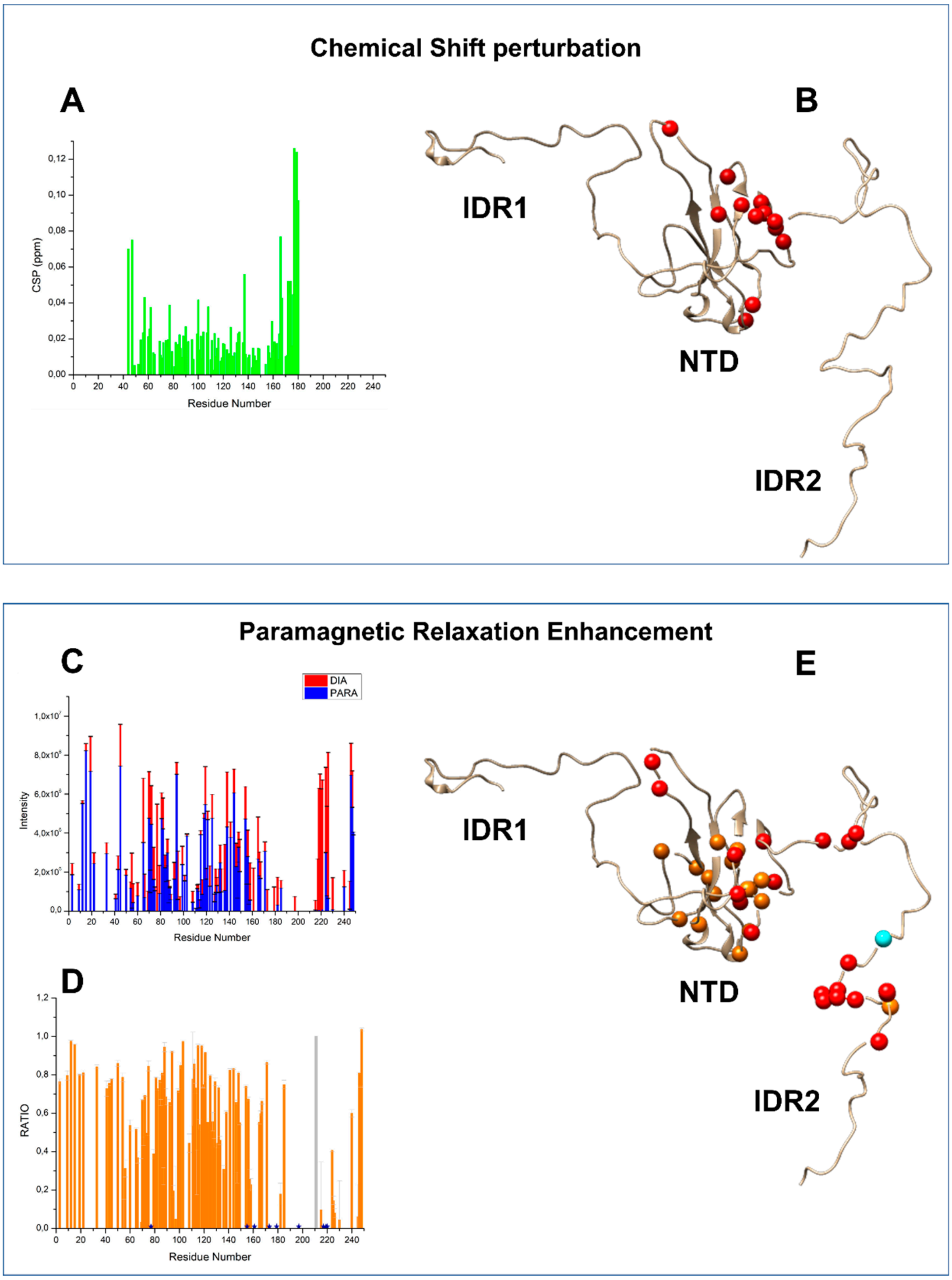

Figure S6: Chemical Shift Perturbation (CSP) and Paramagnetic Relaxation Enhancement (PRE) results.

Panel A reports the CSP values as obtained comparing the chemical shift values of H^N and N resonances of the NTR construct with those of the NTD alone. The residues with a CSP value higher than 0.03 (average + 1 standard deviation) are mapped on a protein model in panel B. The four terminal residues of NTD (44-47 and 177-180) were excluded as their chemical environment, and thus their CSPs, are mainly influenced by the absence vs. presence of the IDR residues themselves.

Panel C reports the intensity values as obtained from the diamagnetic (red) and the paramagnetic (blue) spectra of the A211C mutant. We decided to place the spin label at position 211, prior to the ^{216}DAALALLLLD^{225} region but still quite distant from it to avoid perturbing such a crucial region, and also distant from the SR-rich region (^{177}RGGSQASSRSSSRSRNSSRNSTPGSSR^{203}). The ratio between the two forms was calculated and reported in panel D against the residue number. The asterisks represent the residues whose peaks are broadened beyond detection in the paramagnetic spectrum. This ratio is mapped in panel E on a protein model. The residues displaying an intensity ratio between 0 to 25% are reported in red and those from 25.1% to 50% are reported in orange. The position of the spin label is depicted in cyan.

References

3. Wacker, A.; Weigand, J.E.; Akabayov, S.R.; Altincekic, N.; Bains, J.K.; Banijamali, E.; Binas, O.; Castillo-Martinez, J.; Cetiner, E.; Ceylan, B.; et al. Secondary Structure Determination of Conserved SARS-CoV-2 RNA Elements by NMR Spectroscopy. *Nucleic Acids Res.* **2020**, *48*, 12415–12435, doi:10.1093/nar/gkaa1013.
57. Popenda, M.; Szachniuk, M.; Antczak, M.; Purzycka, K.J.; Lukasiak, P.; Bartol, N.; Blazewicz, J.; Adamiak, R.W. Automated 3D Structure Composition for Large RNAs. *Nucleic Acids Res.* **2012**, *40*, e112–e112, doi:10.1093/nar/gks339.
58. Hofacker, I.L. Vienna RNA Secondary Structure Server. *Nucleic Acids Res.* **2003**, *31*, 3429–3431, doi:10.1093/nar/gkg599.
59. Blom, N.; Sicheritz-Pontén, T.; Gupta, R.; Gammeltoft, S.; Brunak, S. Prediction of Post-Translational Glycosylation and Phosphorylation of Proteins from the Amino Acid Sequence. *Proteomics* **2004**, *4*, 1633–1649, doi:10.1002/pmic.200300771.
